# Evaluation of the neural function of nonhuman primates with spinal cord injury using an evoked potential-based scoring system

**DOI:** 10.1038/srep33243

**Published:** 2016-09-15

**Authors:** Jichao Ye, Mengjun Ma, Zhongyu Xie, Peng Wang, Yong Tang, Lin Huang, Keng Chen, Liangbin Gao, Yanfeng Wu, Huiyong Shen, Yuanshan Zeng

**Affiliations:** 1 Department of Orthopedics, Sun Yat-sen Memorial Hospital, Sun Yat-sen University, Guangzhou, Guangdong 510045, China; 2 Biotherapy Centre, Sun Yat-sen Memorial Hospital, Sun Yat-sen University, Guangzhou, Guangdong 510045, China; 3 Department of Histology and Embryology, Zhongshan School of Medicine, Sun Yat-sen University, Guangzhou, Guangdong 510045, China

## Abstract

Nonhuman primate models of spinal cord injury (SCI) have been widely used in evaluation of the efficacy and safety of experimental restorative interventions before clinical trials. However, no objective methods are currently available for the evaluation of neural function in nonhuman primates. In our long-term clinical practice, we have used evoked potential (EP) for neural function surveillance during operation and accumulated extensive experience. In the present study, a nonhuman primate model of SCI was established in 6 adult cynomologus monkeys through spinal cord contusion injury at T8–T9. The neural function before SCI and within 6 months after SCI was evaluated based on EP recording. A scoring system including somatosensory evoked potentials (SSEPs) and transcranial electrical stimulation-motor evoked potentials (TES-MEPs) was established for the evaluation of neural function of nonhuman primates with SCI. We compared the motor function scores of nonhuman primates before and after SCI. Our results showed that the EP below the injury level significantly changed during the 6 months after SCI. In addition, a positive correlation was identified between the EP scores and motor function. The EP-based scoring system is a reliable approach for evaluating the motor function changes in nonhuman primates with SCI.

Spinal cord injury (SCI) patients often suffer from permanent and irreversible sensorimotor disorders even autonomic functional disturbance. Animal models of SCI play an important role in the investigation of SCI pathophysiology as well as the evaluation of the effectiveness and safety of various restorative interventions^1^. For example, rodent models of SCI have been widely used in many applications, such as the exploration of pathophysiological mechanisms of spinal cord injury, evaluation the effectiveness and safety of experimental treatment via evaluation of motor function and further study of repair mechanism^2^. However, the intervention efficacy in rodent models of SCI may be over-estimated because of highly spontaneous recovery rates, even in serious injuries. The spinal cords of nonhuman primates are anatomically and physiologically similar with that of humans, particularly with respect to the location and function of corticospinal tracts^3^. Studies on complete and partial SCI in nonhuman primates have recently been reported^4^,^5^,^6^,^7^. However, most neural function assessment methods varied which lead to inconsistent evaluation results^8^,^9^,^10^,^11^. Currently, some restorative interventions for SCI patients can only improve sensory disturbances, but have no significant effects on motor function^12^. However, more motor function was included in most evaluation systems, and sensory function was less considered^13^,^14^,^15^.

The standard neurological classification of SCI established by the American Spinal Injury Association (ASIA) is commonly used for neurological evaluation of SCI patients worldwide^16^,^17^,^18^,^19^,^20^,^21^. However, this protocol is not feasible for neurological evaluation of nonhuman primates: assessments of precise muscle strength and sensory tests of key points are unable to be conducted in nonhuman primates because it very difficult to have physical examination with nonhuman primates as same as human beings. Nerve electrophysiological examination, including motor and sensory evoked potential, can reflect integrity and signal transmission of movement and sensory nerve conduction bundles through the changes of latency and amplitude. The motor and sensory evoked potential tests provide an objective assessment method for the evaluation of the motor and sensory conduction bundles in spinal cord, which make it possible to establish a neural functional rating scale in nonhuman primates similar to the ASIA scoring system for SCI patients^22^,^23^.

Cynomologus monkeys are physiologically similar to humans. In addition, a large number of cynomologus monkeys that are available in Guangdong Landao Biotechnology Co. Ltd. allow us to conduct statistical analyses and draw robust conclusion. In the present study, we established a SCI model in cynomologus monkeys and evaluated the sensorimotor function recovery based on the transcranial electrical stimulation-motor evoked potentials (TES-MEPs) and somatosensory evoked potentials (SSEPs) for 6 months after SCI. We also designed a neural functional rating scale based on EP for nonhuman primate models of SCI, which was defined as TES-MEPs score plus SSEPs score.

## Results

All animals survived after SCI surgery and their spinal cords were contused to successfully establish the SCI model. By the first week postoperatively, no monkey needed manual squeeze urine and their involuntary urinary function recovered well. Only one animal was sacrificed in a humane manner at the 10 weeks after SCI surgery because of non-healing skin lesions on the feeder’s advice.

### TES-MEPs, SSEPs and the dynamic changes of the EP scores

TES-MEPs of the abductor pollicis brevis, quadriceps femoris, musculi hippicus, extensor halluces longus, and abductor hallucis were detected and recorded for all monkeys before SCI. The latencies of every animal were slightly different from each other, and the latencies of everyone kept relatively constant in the 4 records before SCI. From abductor pollicis brevis to abductor halluces, along with the extension of the motor transduction pathway, the latencies increased gradually. Compared with the latency, the amplitudes of TES-MEPs were more various. For example, the amplitudes of C1 (animal number) and C2 were different, and the amplitudes of abductor pollicis brevis and abductor halluces of C1 were also different and without rules such as latencies. Even the amplitudes of pollicis brevis in C1 were different every time. We recorded four values of the amplitude of each muscle in each animal before SCI, and the mean value of four records was taken as the baseline of amplitude before SCI (Supplementary Tab. S1 and S2, Fig. S5-a and S5-b).

**Figure 1:**
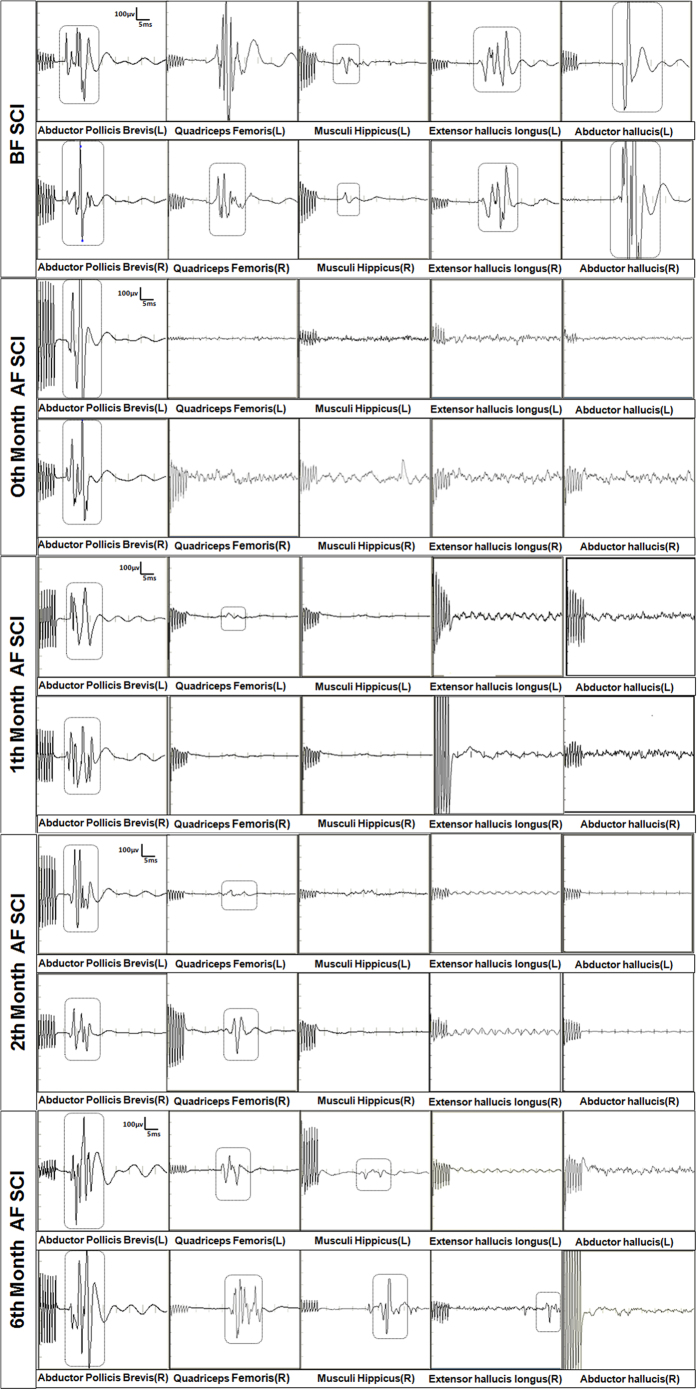
Representative TES-MEPs of C1. Before SCI, the TES-MEPs of all muscles in front and hind limbs were evoked. The TES-MEPs of bilateral hind limbs were not evoked immediately after SCI. The TES-MEPs of bilateral quadriceps femoris were evoked about one month after SCI, but the latency was significantly longer (>10%) than that before SCI and the amplitude significantly decreased (>50%) compared with that before SCI. The TES-MEPs of bilateral gastrocnemius and tibialis anterior muscles were not evoked after SCI. Two months after SCI, the latency was also significantly prolonged and the amplitude was significantly decreased compared with that before SCI, but they were improved step by step. The TES-MEPs of left gastrocnemius were evoked, but the latency was significantly prolonged (>10%) and the amplitude was significantly decreased (>50%). The TES-MEPs of the other muscles were not evoked. Waveforms of evoked potentials were circled in the dashed boxes. “BF SCI” = “Before Spinal Cord Injury”, “AF SCI” = “After Spinal Cord Injury”, “(L)” = “Left limb”, and “(R)” = “Right limb”.

However, the TES-MEPs of lower limb muscles became undetectable soon after SCI. At the end of the 5^th^ week after SCI, the TES-MEPs of lower limb muscles could be detected again, but the amplitude was significantly lower and the latency period was significantly prolonger than the signal detected before SCI. In addition, the amplitude continuously increased until 6 months later when the amplitude and latency period was difficult to restore (Fig. 1).

SSEPs of bilateral median nerves, femoral nerves, tibial nerves, common peroneal nerves, and intercostal nerves from T3 to T12 were detected and recorded for all monkeys before SCI. The latencies of every animal were slightly different from each other, and the latencies of everyone kept relatively constant in the 4 records before SCI. From bilateral median nerves to common peroneal nerves, along with the extension of the sensory transduction pathway, the latencies increased gradually. Compared with the latency, the amplitudes of SSEPs of each nerve were various and with no rules to follow. The amplitudes of different animals varied, and the amplitudes of different muscles in one animal also varied. But the amplitudes of one muscle in the same animal were relatively constant every time. We recorded four values of the amplitude of each muscle in each animal before SCI, and the mean value was taken as the baseline of amplitude before SCI (Supplementary Tab. S3 and S4, Fig. S5-c and S5-d).

However, the SSEPs of nerves below the injury level, including the bilateral lower limb nerves and part of the intercostals nerves, became undetectable soon after SCI. The nerves with disappeared SSEPs signal often started from T8 or T9 to lower limb nerves.

**Figure 2:**
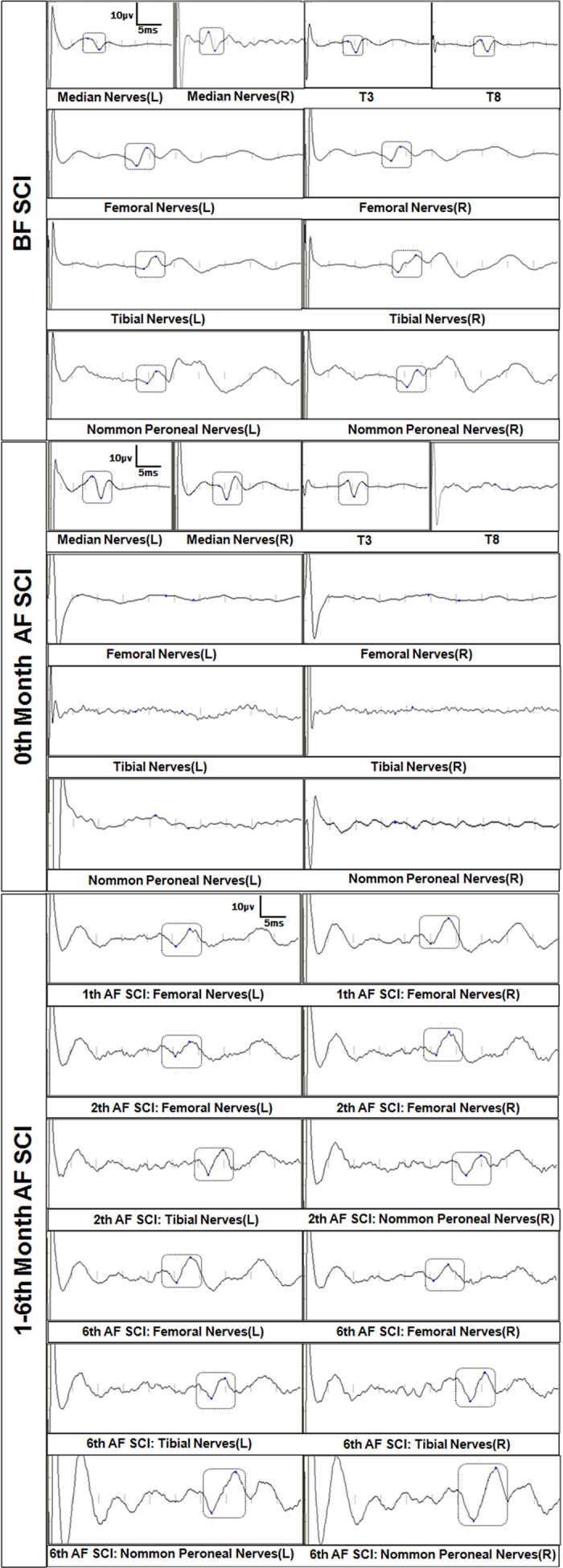
Representative SSEPs of C1. The SSEPs of all nerves were evoked before SCI. The SSEPs of nerves in forelimb and upper intercostal nerves were potentially evoked and the waveforms were evoked, but SSEPs of nerves below the level of injury segment (including bilateral hind legs and lower intercostal nerves) were not evoked. In 1 month after SCI, the SSEPs of several lower intercostal nerves were recovered, the amplitude decreased compared with that before SCI. The SSEPs of bilateral hind limbs nerves were not evoked.

At the end of the 3^th^ week after SCI, the SSEPs of nerves below the injury level were recordable again, but the amplitude was significantly lower and the latency period was significantly prolonger compared to the signal before SCI. The amplitude continuously increased and the latency also continuously shortened until 6 months later, the SSEPs of several nerves could achieve to 50% of the normal amplitude when the increase of latency period was less than 10% of that before SCI (Fig. 2).

The scoring system for neural function consists of 2 factors, the TES-MEPs score and the SSEPs score. For a total of 100 points, TES-MEPs and SSEPS account for 40 (40%) and 60 (60%) points, respectively. The scores before and after SCI were compared to evaluate the neural function. Ten bilateral muscles were included in TES-MEP scoring. The TES-MEP score was defined as 0 if the potential of a muscle was not obviously detected after injury even under the highest stimulation intensity. The score was defined as 2 when the potential amplitude decreased over 80% under the same stimulation intensity, or the latency increased over 10%. The score was defined as 4 when the potential amplitude was normal or decreased less than 80% under the same stimulation intensity. A total of 30 bilateral nerves were included in SSEPs scoring. Similarly, the score was defined as 0 if the potential of a nerve was not obviously detected after injury. The score was defined as 1 when SSEPs could be recorded with typical wave, but the amplitude decreased over 50% or the latency increased over 10%. The score was defined as 2 when the amplitude was normal or decreased less than 50%, and the latency increased less than 10% (Table 1).

**Figure 3:**
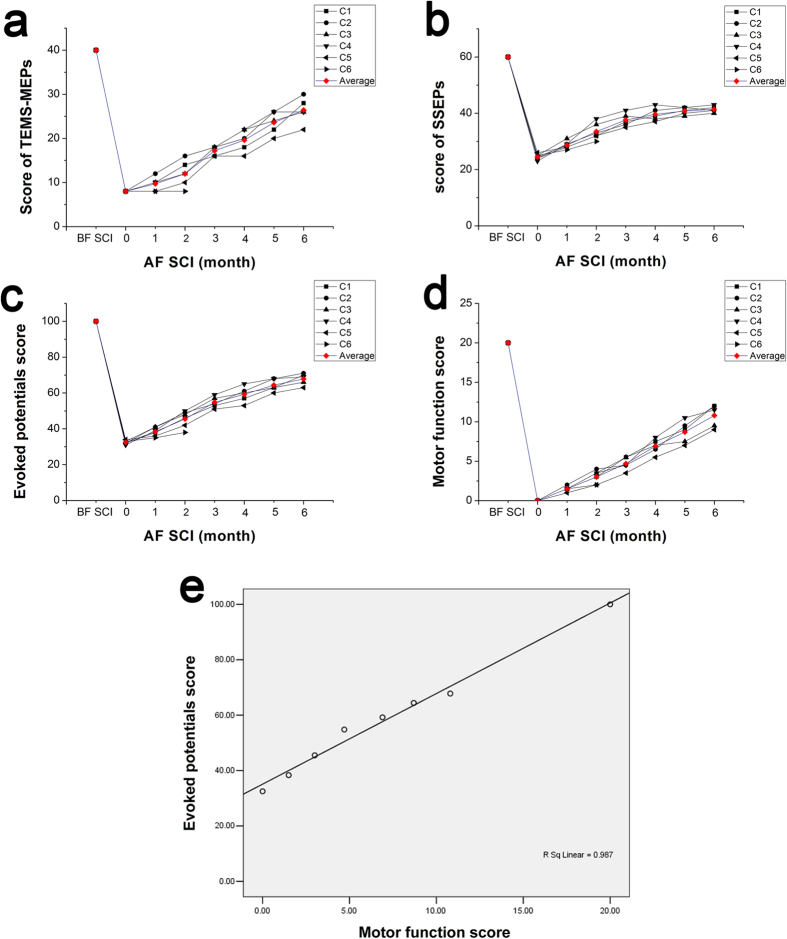
Line charts of the scores. Line charts of the TES-MEP scores (**a**), SSEP scores (**b**), evoked potential scores (**c**), and motor function scores (**d**) before and after SCI (0-6 months). A positive correlation showed between the evoked potential and motor function scores (**e**).

The EP score was defined as TES-MEP score plus SSEPs score. All animals were assigned a full EP score (100 points) before SCI surgery. After SCI, the EP score gradually decreased to minimum (32–34 points). Three to five weeks after SCI, the EP score started to go up due to the increases of the TES-MEP and SSEPs scores. The SSEPs score increased earlier than the TES-MEP score. At the end of the 6^th^ month after SCI, the EP score gradually reached to a platform and was relatively stable. During the whole period after SCI, the recovery of EP score in early phase was faster than the later phase (Fig. 3a–c).

### Neuromotor observation and motor function scores of lower limbs

No movements were observed in lower limbs in the first 4 weeks after SCI surgery. In the early stage after SCI, bilateral lower limbs were flaccid paraplegia without muscle tension. Animals depended on their upper limbs to crawl and their lower limbs were passively pulled without control. Thus, the animals’ palms were always towards the top. When urinary retention appeared, the monkeys’ lower abdomen was compressed for urine. One week after SCI, the tension of lower limb muscles began to recover and then came into spastic paralysis in 2–3 weeks. Bladder function improved much earlier than lower limb muscles, and most animals had automatic micturition within 1 week.

One month after SCI, slight lower limb movements such as hip flexion were observed in several monkeys. Joint activities from knee to ankle joints were gradually observed, and the range of joint flexion in both lower limbs gradually increased. With the joint flexion of lower limbs, pelmas gradually turned to sideways from upwards, and finally turned to downwards. At the end of 8–10 weeks after SCI, animals tried to support the rear body with lower limbs with the recovery of muscle strength of lower limbs, and their buttocks could transiently lift off the ground, which was the most significant event in the entire neural function recovery. With the increase of the muscle strength of lower limbs and pelmas, the animals began to stumble. When climbing mesh fence, the lower extremities could not be controlled at first. Both lower limbs tried to provide supports in the 3^rd^ month after SCI and the force point was mainly on the heel of the foot. With the recovery of lower limb function, the force point gradually transferred to the feet, then to the toes. The motor function scores increased gradually with the recovery of motor function (Fig. 3d).

### The correlation of EP score and ASIA in SCI patients

Preoperative SSEPs of 25 patients with SCI has been evaluated by SSEPs scale (Table 1). Statistic analysis showed that significant correlation between the SSEPs score and sensory score of ASIA (R = 0.902, P = 0.000, Supplementary Fig. S6-a). Postoperative TES-MEPs of 18 patients with SCI has been evaluated by TES-MEPs scale (Table 1). Statistic analysis showed that significant correlation between the TES-MEPs score and motor score of ASIA one week after surgery (R = 0.873, P = 0.000, Supplementary Fig. S6-b).

### Statistic analysis

Firstly, there were statistically significant differences existed in different stages whether EP score or motor score (F = 308.047, P = 0.000). But the recovery pace reflected by EP score and motor score were significantly different (F = 2.928, P = 0.016). And there was no statistically significant difference between the EP and motor function scores based on repeated measures analysis of variance (F = 0.028, P = 0.872).

## Discussion

The recent rapid progress of construction and transportation industries in China has led to a significant increase of the incidence of SCI. Paralysis due to SCI has long-term health, economic, and social consequences^24^,^25^. It has been reported that several interventions such as cell transplantations and Nogo-receptor antibody increased the synaptic plasticity by promoting axonal regeneration and sprouting in rodents with SCI^26^,^27^,^28^. However, there are a number of major differences in the size of nervous systems, neuroanatomical, neurophysiological and behavioral characteristics, and inflammatory and immunological responses between rodents and humans^13^. Therefore, SCI models of nonhuman primates likely provide a better platform than that in rodents for the evaluation of the interventions on human SCI^10^,^11^,^13^. Compared to subjective evaluation of susceptible behaviors, the electrophysiological examination is an objective approach for the evaluation of neural function of nonhuman primates.

Currently, electromyography (EMG) and EP are the main neural electrophysiological methods used in studying SCI models in nonhuman primates^29^. Hernández-Laín *et al*. evaluated the locomotors function of Macaca mulatta before and after complete spinal cord transaction at T8-T9 using EMG^23^. Before SCI, the monkeys were trained to kick a ball with lower limbs and compared with that after SCI. EMG responses were recorded for the animals when kicking the ball for objective evaluation of locomotors function. Under anesthesia, TMS-MEP signals were successfully recorded before SCI and the TMS-MEP signal of lower limbs was not detected soon after SCI. However, the TMS-MEP signal was detected again in 11^th^ week after SCI with prolonged latency period and decreased amplitude. With gradual recovery of the motor function, the latency period decreased, while the amplitude increased. Meng *et al*. investigated the correlation between tibial-somatosensory evoked potentials (T-SEPs) and the locomotors function of paraplegic hind limbs following spinal cord hemi-section in adult rhesus monkeys^30^. T-SEPs were used in this study because it was difficult to steadily record TES-MEPs of the pretibial muscle. They concluded that T-SEPs was an indirect and objective method for assessing the locomotors function in adult monkeys after SCI. We have successfully recorded the SSEPs and TES-MEPs changing before and after SCI. As the motor function recovery, the EP was improved at the same time. What’s more, it seemed like that EP changed earlier than neural function. But the mechanism should be further explored.

EMG measurement needs animals’ cooperation and long-term animal training. Several studies have reported that EMG is not appropriate for evaluating the motor function of nonhuman primates due to poor reliability^11^,^23^,^29^. EP measurement does not rely on animals’ spontaneous activities. The electric or magnetic stimulation in EP measurement directly acts on the head end of the nerve conduction pathway, and signal was recorded in the descending area. The integrity of the neural pathway was determined through the strength of the EP signal and the latency period.

We have extensively used TES-MEPs and SSEPs in clinical practice for the surveillance of neural function during spine operation. In the present study, we applied TES-MEPs and SSEPs to evaluate the neural function in a nonhuman primate model of SCI. Stable and reliable TES-MEPs and SSEPs signal was recorded, suggesting that TES-MEPs and SSEPs are feasible for the evaluation of neural function of nonhuman primate model of SCI. In addition, we established a neural electrophysiology scoring system for several muscles and nerves of both fore-and hind-limbs based on EP amplitude and latency period change before and after SCI. We referred the ASIA score to make ourselves evaluation scale^16^. ASIA has both motor and sensory parts. There are 56 sites for sensory evaluation (28 for each side) and they takes up 112 points in all. And there are 20 muscles for motor evaluation (10 for each side) and they take up another 100 points. Our point’s distribution is similar to ASIA score and also contains the two parts, SSEPs score for sensory function occupied 60 points of 30 peripheral nerves (15 nerves for each side) and TES-MEPs for motor function occupied another 40 points of 10 muscles (5 muscles for each side).

On consideration of which nerves and muscles to be tested, on one hand, we consulted the clinic and chose the nerves or muscles that tested often. On the other hand, also as our rich experience in clinic, SSEPs of intercostal nerves could sensitively reflect the sensory level changing of SCI patients after treatment, especially those with paraplegia. However, the strong intercostal muscles in T1-T2 level always make the SSEPs unstable (the electrodes cannot reach the fixed stimulation site), so we tested the SSEPs of intercostal nerve from T3.

This scoring system provided accurate and reliable evaluation of the neural function of lower limbs because the function of both motor and sensory pathways was rated. Given that the muscles and nerves of upper-limbs were also included in this scoring system with minor modification, this scoring system may be appropriate for evaluating the neural function of cervical SCI models.

EP measurement can be easily interfered. First, Narcotic drugs have significant influences on EP measurement. Inhaled anesthetics have significant inhabitation on cerebral cortex and have obviously inhabitation on SSEPs. Ketamine has a complex effect on the brain, and it was found that the baseline of SSEPs became unstable with Intravenous application of Ketamine in our earlier study. Excessive muscle relaxants can block signal transduction of neuromuscular junction and lead to motor evoked potential non-inducible. We used Propofol for induction (2.5mg/kg, IV) and maintenance (5 mg/kg·h, IV) of anesthesia, and obtain a more stable and reproducible evoked potential. Second, accurately locating and properly fixing the electrodes can avoid electrode shifting or falling off, which also contributed to stable EP measurement. In addition, once we have detected significant potential interference caused by nearby electric hair device used for animal skin preparation. Therefore, turning off electrical equipments in animal operation room as many as possible was important to stable recording of EP signal.

If the interference factors are eliminated and there is no EP signal inducible after SCI, there is no doubt that the score on this project will be 0. But when EP signal were described after SCI, how to determine the evoked potential is normal or abnormal is a worth considering problem. Whether the EP is normal or not is judged by its latency and amplitude. As it was described in the result part, the latencies of SSEPs and TMS-MEPs kept relatively constant in the 4 records before SCI and latencies increased gradually along with the extension of the transduction pathway. So we considered that the warning criteria of SSEPs is amplitude decreased more than 50% or latency increased more than 10% according to the clinic work^31^,^32^,^33^,^34^,^35^,^36^, and then only half of the normal score was obtained. Similarly based on the clinic experience, we made the warning criteria of TES-MEPs “all or none”^37^. When the amplitude of TES-MEPs decreased more than 80%, it may indicate that probably the neural pathway is injured already^38^. So we considered amplitudes of TES-MEPs reduced over 80% contrast to the baseline value as exceptions, then only half of the normal score was obtained.

EP recordings performed in humans over one year after an SCI have enabled to allow an early prediction about the functional outcome (e.g. locomotors ability) after a traumatic injury^39^,^40^. We analyzed the relationship between EP score and ASIA score in SCI patients and found they were statistically correlated. Nevertheless, it still needs continuous observation and further study to explore whether the application of neurophysiological evaluating neural function in clinic is appropriate.

In the 3rd month after SCI, an animal exhibited paroxysmal tic of limbs with more serious manifestations in upper limbs when recording the TES-MEPs. This issue has been rarely reported. The electroencephalogram showed electrical disorders and abnormal discharge. We considered this incidence as epileptic seizure caused by transcranial electrical stimulation. TES-MEPs recording was immediately terminated. Intravenous administration of animal diazepam (1mg/kg) was used to strengthen insulation, which finally released the tic.

## Materials and Methods

### Animal Subjects

All the humans and animal experiments in the present study were approved and supervised by the Animal Care and Ethical Committee of Sun Yat-sen University (Guangzhou, China). All the human and animal studies have been performed in accordance with the ethical standards laid down in the 1964 Declaration of Helsinki and its later amendments. Informed consent was obtained from all individual participants included in the study. The methods were carried out in accordance with the approved guidelines. 6 male and adult cynomologus monkeys of 4-5 years and 4.6–6.2 kg of weight were included. All animals were provided by Guangdong Landao Biotechnology Co. Ltd (Guangzhou, China) and housed individually in stainless steel cages. A mirror was attached to the outside of cages to allow animals to view activities in most of the room. Natural lighting time lasted for 12 hours daily from 7:00 a.m. to 7:00 p.m. All animals received a regular diet containing high fat forage (150 gram) and green fodder (20–50 gram) every day. Fasting started at 4:00 p.m. on the day before blood sampling, operation, or anatomy. Automatic water dispensers are available for animals and enrichments including forage boards, chew toys, and radio were provided daily.

### TES-MEPs and SSEPs

TES-MEPs and SSEPs of each cynomologus monkey were recorded every week before SCI for 4 weeks to obtain the baseline of potentials, including the normal range of the amplitude and latency of TES-MEPs and SSEPs when the nervous system is normal. And TES-MEPs and SSEPs were also recorded after SCI (details of SCI model was described in 2.3) immediately and every month after SCI for 6 months. The potentials were record with the Nicolet Viking IV evoked potential monitors according to the manufacture’s instruction (Nicolet Biomedical, Madison, WI, USA) and needle electrodes were used as stimulating and recording electrodes. When recording TES-MEPs and SSEPs, anesthesia was induced with Propofol (2.5mg/kg, IV, ASTRAZENECA PLC, UK) and maintained with Propofol (5 mg/kg·h, IV). Oxygen flow (1.5 L/min) delivered through an endotracheal tube. The hair on head, chest-abdomen, and limbs was scraped off.

**Figure 4:**
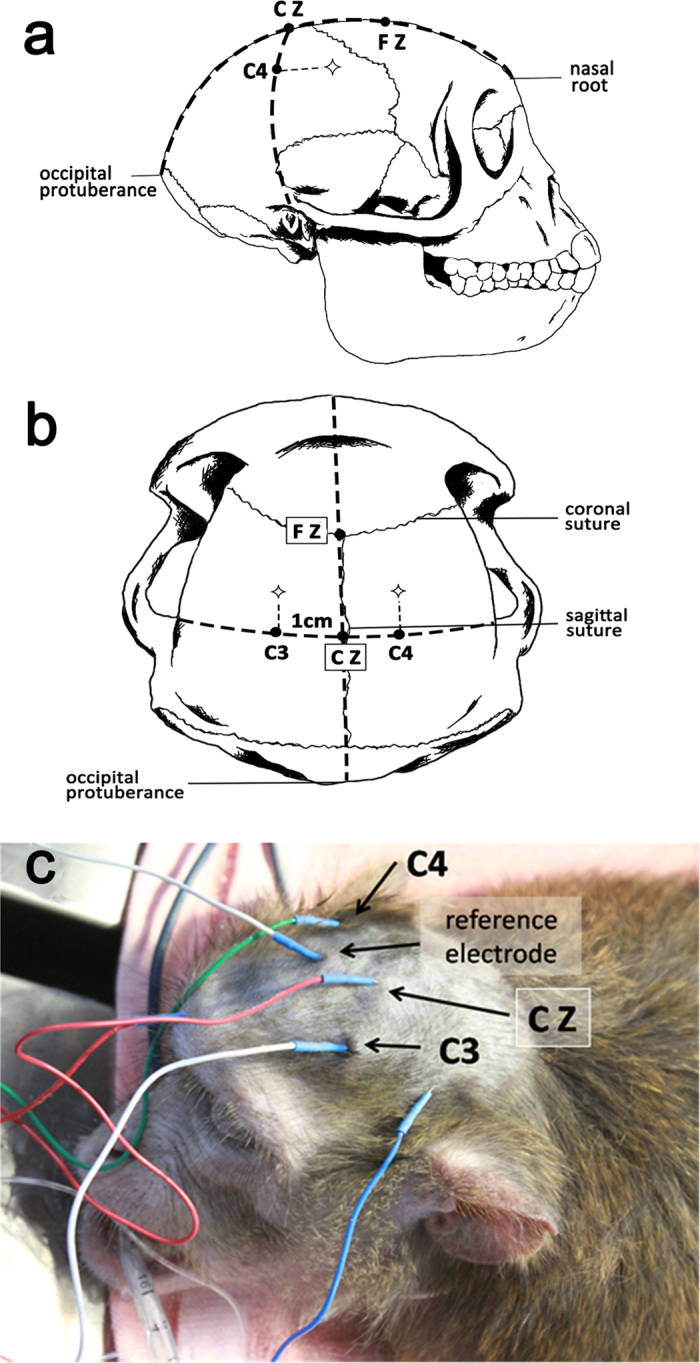
The location of electrodes inserted and the stimulating area. (**a**) The anterior-posterior (AP) sagittal line: made a line from the nasal root to the external occipital protuberance and took five points formerly backward, in turn named as Fpz, Fz, Cz, Pz and Oz. The distances of the Fpz to the nasal root and the Oz to the external occipital protuberance respectively comprised about 10% of this line and the others were equality apart by 20% of the line. (**b**) The transverse line: made another line from the left pre-auricular to the right pre-auricular which intersected the AP sagittal line at Cz. Then we took two points on each side of the transverse line, T3 (Not marked in the picture) and C3 on the left, T4 (Not marked in the picture) and C4 on the right. The distances of T3 to the left pre-auricular and T4 to the right pre-auricular respectively comprised about 10% of the transverse line and the others were equality apart by 20% of the line (include the point Cz). (**c**) As this actual picture showed that during the TES-MEPs monitoring the stimulating electrodes placed about 1 cm in front of C3 and C4, they were each other’s reference electrode. During the SSEPs monitoring, the receiving electrode placed at Cz and the reference point placed at random point.

To record TES-MEPs, according to the international 10/20 systems provided by the International Electroencephalographic Society, the stimulating electrodes were placed 1cm anterior to C3/C4 (Fig. 4), and the each other was consider as a stimulating electrode and reference electrode. Short train transcranial electrical stimulation was initiated at 7 square-waves from the anode, 300 μs duration, 1ms interstimulus interval (1000Hz repetition frequency), and 40V stimulation voltage. Myogenic MEPs of abductor pollicis brevis, quadriceps femoris, musculi hippicus, extensor halluces longus, and abductor hallucis were recorded.

To record SSEPs, needle electrodes and metal strip electrodes were used as recording and stimulating electrodes, respectively. The reference electrodes were placed at the Fz point and the recording electrodes were set at the Cz point (Fig. 4). The stimulating electrodes were placed on the bilateral median nerves in upper limbs, intercostals nerves from T3 to T12 in axillary midline of chest wall, femoral nerves, tibial nerves, and the common peroneal nerves in lower limbs. The stimulating intensity was 20 mA, the stimulating frequency was set at 3.1Hz, and the wave band ranged from 50–300 Hz. The analysis time started from 50ms until obtained a straight baseline. The location of intercostal nerves depended on the sternum, ribs, and other anatomical structures. According to the nerve segmental distribution of human torso and the feasibility of SSEPs, a total of 10 (from T3–T12) intercostal nerves were detected.

**Figure 5:**
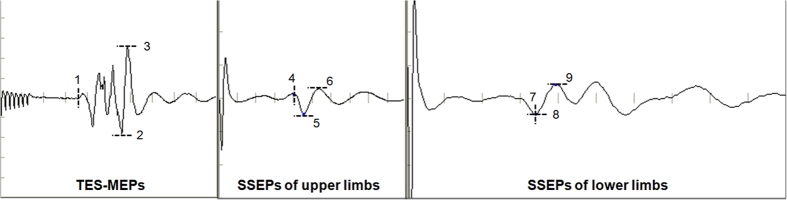
Latency and amplitude measurement of TES-MEPs and SSEPs. the stimulus to the first major negative peak (upward deflection, marked with number 4 in the SSEPs tracings) in waveform of bilateral median nerves and intercostals nerves or the first major positive peak (downward deflection, marked with number 7 in the SSEPs tracings) in the waveform of nerves located in lower limbs. The amplitude (μv) was measured from the major negative peak (upward deflection, marked with number 6/9 in the SSEPs tracings) to the major positive peak (downward deflection, marked with number 5/8 in the SSEPs tracings)

The measurement methods of latency and amplitude of TES-MEPs and SSEPs were shown in Fig. 5.

### Establishment of the SCI model in cynomologus monkeys

**Figure 6:**
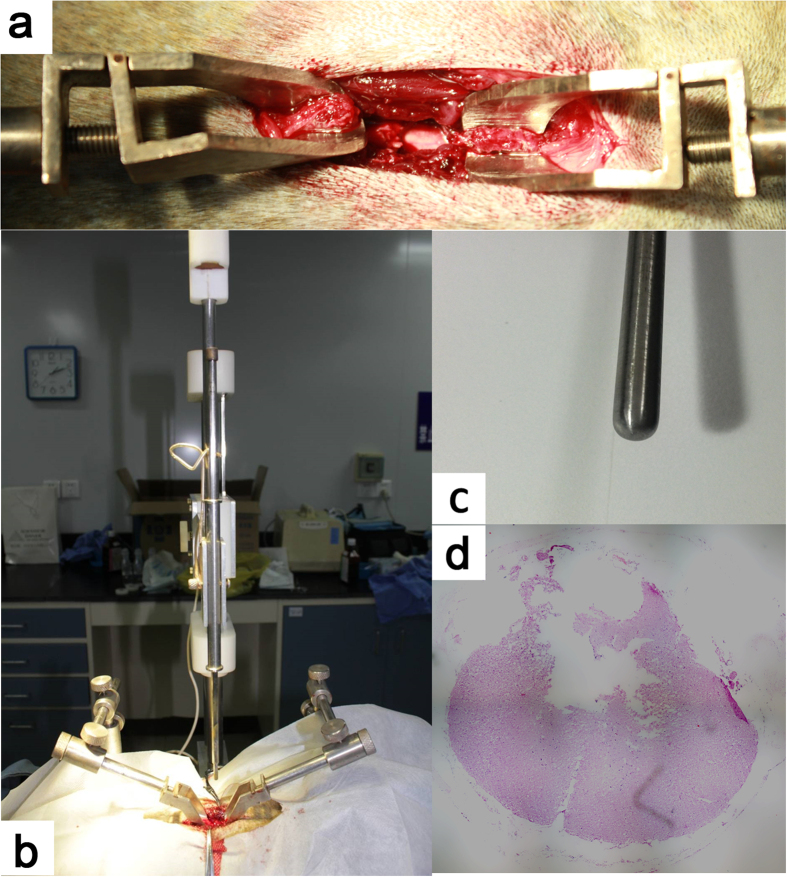
Establishment of the SCI model. (**a**)We surgically removed the thoracic vertebral laminae to expose the spinal cord and then fixed the spinous process with two fixtures. (**b**) We fixed the animal on the SS-II spinal cord contusion impactor that was prepared to creat the model. (**c**) A close-up of the striker. It is a blunt cylinder with 6 mm diameter. (**d**) The extent of contusion lesion at T8-T9 spinal segment. 6 months after SCI, we transected the contusive segment of the spinal cord to make HE staining that showed the syringe myelia had been formed.

Anesthesia was induced with Propofol (2.5 mg/kg, IV, ASTRAZENECA PLC, UK) and maintained with Propofol (5 mg/kg·h, IV) and Remifentanil of (0.3 mg/kg·h, Renfu Pharmaceutical Co., Ltd., Yichang, China). Oxygen flow (1.5L/min) delivered through an endotracheal tube. Animals received prophylactic antibiotic Ceftriaxone Sodium (50 mg/kg/24 h, im, Rocephin^®^, Roche Pharmaceutical Co., Ltd., Shanghai, China) within 72h after surgery. To establish the SCI model, the spinal processes and vertebral laminae of T6–7 were surgically removed and the T8–T9 spinal cord located around the T6–T7 vertebrae. When the endo rhachis was revealed well, the spinal cord contusive injury was induced with SS-II spinal cord contusion impactor^41^ with a 50 g weight away from the fall to 50.0 mm height (Fig. 6). Animals were monitored for vital signs according to the standard post-anesthesia care for humans. Neurologic examination was performed after preliminary extubation and anesthesia. After recovered from anesthesia, the animals were sent back to their cages with a mattress placed on the bottom to minimize the risk of pressure sore. Animals were observed twice a day to assess skin integrity and exclude the possibility of autophagy, which can be observed in the setting of limb denervation.

### Neuromotor video recording and evaluation of motor function

During the study, in order to analysis the correlation between evoked potentials (TES-MEPs and SSEPs) and motor function pre-SCI or post-SCI, we scored the motor function via a score table (Table 2) which had been reported in literature for evaluating non-human primate lower limb motor function after SCI^8^.

The neuromotor function was evaluated based on video recording and rating of the voluntary movements of the monkeys’ lower limbs (Pritchard *et al*. 2010). Video recording of monkeys’ locomotion was obtained with a camera placed in a foot chamber (8cm × 5cm× 5cm), one side of which was made by Plexiglas. Monkeys’ movements were recorded continuously for 30 minutes each time. During this period, food rewarding was introduced to the chamber through an aperture in the ceiling to promote and document up right standing for several times. Videos were recorded before SCI and weekly for a total of 24 weeks postoperatively. The video data were reviewed and rated by two blinded reviewers who had not participated in the early stage of the present study. A comprehensive neuromotor function scoring system was generated on the basis of the ratings for both ipsilateral and contralateral hind limbs movements (Table 2).

Before exsanguination execution, the animal was heavily doped with pentobarbital sodium (0.5ml/kg) (Shanghai National Medicine Co. Ltd., batch number: 120205) via saphena vein injection. Deep anesthesia was reached when the monkey had no responses. The animal body was burned after anatomy.

Repeated measures analysis of variance was conducted to evaluate the relationship between the EP score and the motor function score (P < 0.05).

### The correlation of EP score and ASIA score in SCI patients

SSEPs: 25 SCI patients in total, 19 males and 6 females; 18 cases with acute injury (have passed through the spinal cord shock period) and 7 cases with chronic injury. Preoperative SSEPs of 25 patients were evaluated by SSEPs scale (Table 1). And then we analyzed the correlation of EP score and ASIA sensory score over the same period with Pearson correlation coefficient (P < 0.001).

TES-MEPs: 18 SCI patients in total, 13 males and 5 females; 12 cases with acute injury (have passed through the spinal cord shock period) and 6 cases with chronic injury. Postoperative TES-MEPs of 18 patients with SCI has been evaluated by TES-MEPs scale (former part of Table 1). And then we analyzed the correlation of EP score and ASIA motor score within one week after surgery with Pearson correlation coefficient (P < 0.001).

## Additional Information

**How to cite this article**: Ye, J. *et al*. Evaluation of the neural function of nonhuman primates with spinal cord injury using an evoked potential-based scoring system. *Sci. Rep.* **6**, 33243; doi: 10.1038/srep33243 (2016).

## Supplementary Material

Supplementary Information

## Figures and Tables

**Table 1:** Evoked potential score of thoracic spinal cord injury in nonhuman primates.

TES-MEPs						
	No TES-MEPs		Yes, but Amplitude decreased over 50%; or latency increase over 10%		Normal OR Amplitude decreased below 50% and latency increase below 10%	
Target muscles	Left	Right	Left	Right	Left	Right
Abductor Pollicis Brevis	0	0	2	2	4	4
Quadriceps Femoris	0	0	2	2	4	4
Musculi Hippicus	0	0	2	2	4	4
Extensor Hallucis Longus	0	0	2	2	4	4
Abductor Hallucis	0	0	2	2	4	4
Total						
**SSEPs**						
	**No SSEPs**		**Yes, but Amplitude decreased over 50%; or latency increase over 10%**		**Normal OR Amplitude decreased below 50% and latency increase below 10%**	
**Target nerves**	**Left**	**Right**	**Left**	**Right**	**Left**	**Right**
Bilateral median N	0	0	1	1	2	2
Bilateral median N	0	0	1	1	2	2
T3	0	0	1	1	2	2
T4	0	0	1	1	2	2
T5	0	0	1	1	2	2
T6	0	0	1	1	2	2
T7	0	0	1	1	2	2
T8	0	0	1	1	2	2
T9	0	0	1	1	2	2
T10	0	0	1	1	2	2
T11	0	0	1	1	2	2
T12	0	0	1	1	2	2
Femoral N	0	0	1	1	2	2
Tibial N	0	0	1	1	2	2
Nommon Peroneal N	0	0	1	1	2	2
Total						

The EP-based scoring system contained two components, the TES-MEPs and SSEPs. The total EP score was 100 points, including 40% of TES-MEPs and 60% of SSEPs. A total of 10 bilateral muscles were included in the scoring of TES-MEPs. The SSEPs and TES-MEPs scores before and after SCI were compared to evaluate the neural function of nonhuman primates. Contrast with the initial EP scores before SCI, the changes of EP scores after SCI were divided into three levels: ① 0 point when no EP was recorded; ② when SSEPs was recorded, but the amplitude decreased over 50% or the latency increased over 10% compared to base data,1 point of SSEPs was got; when TES-MEPs was recorded, but the amplitude decreased over 80% or the latency increased over 10% compared to base data, 2 point of TES-MEPs was got; ③ when SSEPs was recorded, the amplitude was higher/normal/decreased less than 50%, or the latency was shorten/normal/prolonged less than 10% compared to base data, 2 point of SSEPs was got; when TES-MEPs was recorded, the amplitude was higher/normal/decreased less than 80%, or the latency was shorten/normal/prolonged less than 10% compared to base data,4 point of TES-MEPs was got. If the amplitude and latency of EP of upper limbs were significantly changed, then we recommend ratio of lower extremity potential and upper extremity potential as evaluation criteria.

**Table 2:** Ambulation chamber video observational neuromotor score.

Score	Description
0	No voluntary function
1	Slight movement of one or two joints
2	Active movement of one or two joints, slight movement of other joints
3	Active movement of all three joints, no weight bearing
4	Slight weight bearing, consistent dorsal stepping (no plantar stepping)
5	Slight weight bearing, occasional plantar stepping
6	Frequent plantar stepping, occasional weight bearing, hops with partial weight support
7	Frequent plantar stepping and weight bearing, occasional FL-HL coordination
8	Consistent plantar stepping and partial weight supported steps, occasional FL-HL coordination
9	Frequent partial weight supported steps, occasional FL-HL coordination
10	Occasional partial weight supported steps, frequent foot drop and/or drag, run with partial weight support
11	Occasional partial weight supported steps, frequent FL-HL coordination
12	Slight partial weight supported steps, frequent FL-HL coordination, stands up HL with partial weight support
13	Slight partial weight supported steps, consistent FL-HL coordination, frequent foot drop and/or drag
14	Full weight supported steps and consistent FL-HL coordination, occasional foot drop and/or drag
15	Occasional foot drop and/or drag, stand up HL with full weight support
16	Slight foot drop and/or drag, no toe clearance
17	No foot drop and/or drag, no toe clearance
18	Occasional toe clearance
19	Frequent toe clearance
20	Normal

The animals were placed in an open flat ground for behavior observation; 2 observers scored for animals. The observation was more than 15 minutes, and the video was recorded at the same time. Score should been played in the morning and urinate was need before the score.
