# Supplementary material for: Evaluation of the neural function of nonhuman primates with spinal cord injury using an evoked potential-based scoring system
Source: Sci Rep. 2016 Sep 15;6:33243. doi: 10.1038/srep33243 (PMC5024084; doi:10.1038/srep33243)
Supplement: Supplementary Information [file srep33243-s1.doc]

**Evaluation of the neural function of nonhuman primates with spinal cord injury using an evoked potential-based scoring system**

**Jichao Ye^a, +^, Mengjun Ma^a, +^, Zhongyu Xie^a^, Peng Wang^a^, Yong Tang^a^, Lin Huang^a^, Keng Chen^a^, Liangbin Gao^a^, Yanfeng Wu^b^, Huiyong Shen^a,*^, and Yuanshan Zeng^c^**

^a^ Department of Orthopedics, Sun Yat-sen Memorial Hospital, Sun Yat-sen University, Guangzhou, Guangdong 510045, China

^b^ Biotherapy Centre, Sun Yat-sen Memorial Hospital, Sun Yat-sen University, Guangzhou, Guangdong 510045, China

^c^ Department of Histology and Embryology, Zhongshan School of Medicine, Sun Yat-sen University, Guangzhou, Guangdong 510045, China

^*^ Corresponding author at: Department of Orthopedics, Sun Yat-sen Memorial Hospital, Sun Yat-sen University, 107#, Yanjiang Xi Road, Guangzhou, Guangdong 510045, China. Tel.: +86 020 81332032; Fax: +86 020 81332319; Email: shenhuiyong@aliyun.com

^+^These authors contributed equally to this work.

| Time Muscle | BF SCI (week 1) | | BF SCI (week 2) | | BF SCI (week 3) | | BF SCI (week 4) | | Avergae (basic value) of week1-4 | |
| --- | --- | --- | --- | --- | --- | --- | --- | --- | --- | --- |
|  | L(ms) | A(μv) | L(ms) | A(μv) | L(ms) | A(μv) | L(ms) | A(μv) | L(ms) | A(μv) |
| Abductor Pollicis Brevis (L) | 10.50 | 610.0 | 10.10 | 240.0 | 10.30 | 690.00 | 9.90 | 110.00 | 10.20±2.78 | 412.5±304.4 |
| Abductor Pollicis Brevis (R) | 10.30 | 820.0 | 10.20 | 350.0 | 10.60 | 580.00 | 10.20 | 90.00 | 10.33±2.83 | 460.0±338.9 |
| Quadriceps Femoris (L) | 15.10 | 1050 | 15.00 | 80.00 | 15.20 | 120.00 | 14.80 | 370.00 | 15.03±4.93 | 405.0±428.0 |
| Quadriceps Femoris (R) | 15.00 | 390.0 | 15.10 | 120.0 | 15.30 | 190.00 | 14.60 | 290.00 | 15.00±4.93 | 247.5±149.3 |
| Musculi Hippicus (L) | 16.80 | 190.0 | 16.60 | 330.0 | 16.60 | 220.00 | 16.00 | 300.00 | 16.50±6.00 | 260.0±127.9 |
| Musculi Hippicus (R) | 17.00 | 90.00 | 16.70 | 410.0 | 16.90 | 310.00 | 16.20 | 420.00 | 16.70±5.69 | 307.5±189.9 |
| Extensor halluces longus (L) | 18.70 | 510.0 | 18.30 | 280.0 | 18.80 | 490.00 | 17.90 | 690.00 | 18.43±6.64 | 429.5±262.4 |
| Extensor halluces longus (R) | 18.40 | 530.0 | 18.10 | 250.0 | 19.00 | 610.00 | 18.20 | 530.00 | 18.43±6.46 | 480.0±253.0 |
| Abductor halluces (L) | 22.60 | 1020 | 22.70 | 400.0 | 22.90 | 910.00 | 22.00 | 690.00 | 22.55±8.30 | 755.0±411.0 |
| Abductor halluces (R) | 22.50 | 1120 | 22.40 | 410.0 | 23.10 | 730.00 | 22.40 | 450.00 | 22.60±8.32 | 677.5±413.8 |
| Time Muscle | AF SCI (0th month) | | AF SCI (1th month) | | AF SCI (2th month) | | AFSCI(3th month) | | AF SCI (6th month) | |
|  | L(ms) | A(μv) | L(ms) | A(μv) | L(ms) | A(μv) | L(ms) | A(μv) | L(ms) | A(μv) |
| Abductor Pollicis Brevis (L) | 10.20 | 1230 | 10.90 | 420.0 | 10.80 | 680.00 | 10.70 | 310.00 | 10.00 | 910.00 |
| Abductor Pollicis Brevis (R) | 10.00 | 850.0 | 10.20 | 450.0 | 10.30 | 350.00 | 10.20 | 530.0 | 10.40 | 1010.00 |
| Quadriceps Femoris (L) | — | — | 21.60 | 25.00 | 20.10 | 20.00 | 19.50 | 50.00 | 19.90 | 270.00 |
| Quadriceps Femoris (R) | — | — | — | — | 22.90 | 330.00 | 20.90 | 210.00 | 21.80 | 480.00 |
| Musculi Hippicus (L) | — | — | — | — | — | — | — | — | 23.40 | 78.00 |
| Musculi Hippicus (R) | — | — | — | — | — | — | 35.20 | 55.00 | 25.90 | 490.00 |
| Extensor halluces longus (L) | — | — | — | — | — | — | — | — | — | — |
| Extensor halluces longus (R) | — | — | — | — | — | — | — | — | 43.50 | 130.00 |
| Abductor halluces (L) | — | — | — | — | — | — | — | — | — | — |
| Abductor halluces (R) | — | — | — | — | — | — | — | — | — | — |

**Supplementary Table S1. The latency and amplitude of TES-MEPs of No. C1**

“—” means that no TES-MEPs was recorded. “BF SCI” = “Before Spinal Cord Injury”, “AF SCI” = “After Spinal Cord Injury”; “(L)” = “Left limb”, “(R)” = “Right limb”; "L (ms)"=TES-MEPs latency, "A (μv)"= TES-MEPs amplitude

| **NO. Muscle** | **C1** | | **C2** | | **C3** | | **C4** | | **C5** | | **C6** | |
| --- | --- | --- | --- | --- | --- | --- | --- | --- | --- | --- | --- | --- |
|  | L(ms) | A(μv) | L(ms) | A(μv) | L(ms) | A(μv) | L(ms) | A(μv) | L(ms) | A(μv) | L(ms) | A(μv) |
| Abductor Pollicis Brevis (L) | 10.20 | 412.5 | 10.93 | 87.54 | 10.01 | 690.0 | 9.93 | 473.5 | 11.23 | 443.7 | 10.41 | 324.9 |
| Abductor Pollicis Brevis (R) | 10.33 | 460.0 | 10.97 | 343.9 | 10.20 | 580.0 | 10.06 | 98.05 | 11.37 | 251.0 | 10.53 | 521.3 |
| Quadriceps Femoris (L) | 15.03 | 405.0 | 16.02 | 277.3 | 14.43 | 120.0 | 14.10 | 351.9 | 17.01 | 356.7 | 15.72 | 455.3 |
| Quadriceps Femoris (R) | 15.00 | 247.5 | 15.94 | 328.5 | 14.31 | 190.0 | 13.96 | 211.6 | 16.83 | 491.2 | 15.92 | 362.1 |
| Musculi Hippicus (L) | 16.50 | 260.0 | 17.50 | 480.7 | 16.23 | 220.0 | 16.00 | 492.9 | 18.89 | 433.8 | 17.02 | 321.6 |
| Musculi Hippicus (R) | 16.70 | 307.5 | 17.65 | 406.2 | 16.12 | 310.0 | 16.10 | 381.3 | 18.65 | 525.6 | 17.14 | 504.2 |
| Extensor halluces longus (L) | 18.43 | 429.5 | 19.52 | 534.1 | 18.01 | 490.0 | 17.53 | 402.4 | 19.98 | 411.0 | 19.13 | 320.2 |
| Extensor halluces longus (R) | 18.43 | 480.0 | 18.94 | 613.4 | 17.93 | 610.0 | 17.75 | 544.8 | 20.14 | 441.1 | 18.68 | 283.7 |
| Abductor halluces (L) | 22.55 | 755.0 | 23.78 | 463.6 | 21.66 | 910.0 | 20.95 | 521.9 | 24.32 | 242.9 | 22.78 | 305.2 |
| Abductor halluces (R) | 22.60 | 677.5 | 23.45 | 298.2 | 21.79 | 730.0 | 20.88 | 354.6 | 24.48 | 221.6 | 22.42 | 479.5 |

**Supplementary Table S2. The average (basic value) of TES-MEPs latency and amplitude of 6 animals before SCI**

“C1-C6”is the animal number; “(L)” = “Left limb”, “(R)” = “Right limb”; "L (ms)"=TES-MEPs latency, "A (μv)"= TES-MEPs amplitude

| Time Nerves(N) | BF SCI (week 1) | | BF SCI (week 2) | | BF SCI (week 3) | | BF SCI (week 4) | | Avergae (basic value) of week1-4 | |
| --- | --- | --- | --- | --- | --- | --- | --- | --- | --- | --- |
|  | L(ms) | A(μv) | L(ms) | A(μv) | L(ms) | A(μv) | L(ms) | A(μv) | L(ms) | A(μv) |
| Bilateral median N (L) | 8.10 | 7.10 | 8.10 | 10.40 | 8.30 | 12.10 | 8.60 | 9.10 | 8.28±0.20 | 9.68±1.83 |
| Bilateral median N (R) | 8.30 | 10.10 | 8.20 | 10.30 | 8.60 | 10.40 | 8.20 | 7.40 | 8.32±0.16 | 9.55±1.25 |
| T3 (L) | 8.20 | 6.10 | 8.15 | 7.50 | 8.20 | 9.10 | 8.80 | 7.10 | 8.34±0.27 | 7.45±1.08 |
| T3 (R) | 8.25 | 7.20 | 8.20 | 7.70 | 8.30 | 7.85 | 8.60 | 8.85 | 8.34±0.16 | 7.9±0.60 |
| T8 (L) | 9.80 | 6.00 | 10.00 | 4.50 | 9.60 | 6.50 | 10.00 | 9.50 | 9.85±0.17 | 6.63±1.82 |
| T8 (R) | 9.70 | 4.10 | 9.75 | 5.30 | 9.90 | 8.25 | 10.20 | 8.10 | 9.89±0.19 | 6.43±1.79 |
| Femoral N (L) | 17.20 | 7.70 | 18.30 | 8.20 | 17.50 | 4.90 | 17.90 | 6.90 | 17.70±0.41 | 6.93±1.26 |
| Femoral N (R) | 17.10 | 6.40 | 17.10 | 7.10 | 17.65 | 6.35 | 18.20 | 6.15 | 17.51±0.46 | 6.50±0.36 |
| Tibial N (L) | 18.60 | 5.30 | 19.70 | 6.60 | 18.90 | 7.20 | 19.00 | 6.40 | 19.05±0.40 | 6.38±0.67 |
| Tibial N (R) | 18.50 | 7.45 | 19.40 | 8.40 | 19.10 | 5.90 | 18.40 | 7.60 | 18.85±0.41 | 7.34±0.91 |
| Common peroneal N (L) | 19.70 | 5.10 | 20.00 | 5.50 | 20.20 | 7.15 | 19.80 | 5.85 | 19.93±0.19 | 5.90±0.77 |
| Common peroneal N (R) | 20.10 | 7.60 | 19.90 | 5.90 | 20.05 | 6.20 | 20.15 | 6.90 | 20.05±0.09 | 6.65±0.66 |
| Time Nerves (N) | AF SCI (0^th^ month) | | AF SCI (1^th^ month) | | AF SCI (2^th^ month) | | AFSCI(3^th^ month) | | AF SCI (6^th^ month) | |
|  | L(ms) | A(μv) | L(ms) | A(μv) | L(ms) | A(μv) | L(ms) | A(μv) | L(ms) | A(μv) |
| Bilateral median N (L) | 8.75 | 11.05 | 8.50 | 10.05 | 8.35 | 9.35 | 8.40 | 11.50 | 8.25 | 10.35 |
| Bilateral median N (R) | 8.60 | 11.00 | 8.20 | 9.35 | 8.65 | 8.30 | 8.35 | 10.70 | 8.35 | 7.55 |
| T3 (L) | 8.65 | 7.30 | 8.30 | 8.20 | 8.65 | 7.45 | 8.50 | 8.85 | 8.30 | 8.55 |
| T3 (R) | 8.50 | 7.10 | 8.35 | 6.40 | 8.55 | 8.80 | 8.35 | 7.65 | 8.45 | 7.65 |
| T8 (L) | — | — | — | — | 28.80 | 3.35 | 26.10 | 4.8 | 23.40 | 7.40 |
| T8 (R) | — | — | 27.50 | 3.10 | 24.10 | 4.20 | 25.20 | 5.40 | 25.90 | 6.85 |
| Femoral N (L) | — | — | 25.75 | 5.05 | 25.30 | 4.10 | 25.40 | 5.60 | 25.15 | 7.70 |
| Femoral N (R) | — | — | 25.10 | 7.95 | 25.80 | 6.15 | 24.95 | 4.65 | 26.10 | 5.05 |
| Tibial N (L) | — | — | — | — | — | — | — | — | 32.25 | 6.05 |
| Tibial N (R) | — | — | — | — | 31.05 | 7.60 | 33.05 | 8.80 | 32.50 | 9.05 |
| Common peroneal N (L) | — | — | — | — | 32.10 | 4.90 | 31.90 | 9.90 | 31.75 | 12.45 |
| Common peroneal N (R) | — | — | — | — | — | — | — | — | 33.05 | 15.05 |

**Supplementary Table S3. The latency and amplitude of SSEPs of No. C1**

“C1-C6”is the animal number; “(L)” = “Left limb”, “(R)” = “Right limb”; "L (ms)"=SSEPs latency, "A (μv)"= SSEPs amplitude

| NO. Muscle | C1 | | C2 | | C3 | | C4 | | C5 | | C6 | |
| --- | --- | --- | --- | --- | --- | --- | --- | --- | --- | --- | --- | --- |
|  | L(ms) | A(μv) | L(ms) | A(μv) | L(ms) | A(μv) | L(ms) | A(μv) | L(ms) | A(μv) | L(ms) | A(μv) |
| Bilateral median N (L) | 8.28 | 9.68 | 8.36 | 5.42 | 8.13 | 7.38 | 7.53 | 7.73 | 8.84 | 6.91 | 8.23 | 6.37 |
| Bilateral median N (R) | 8.32 | 9.55 | 8.44 | 6.32 | 8.19 | 6.91 | 7.98 | 7.03 | 8.61 | 7.26 | 8.41 | 5.44 |
| T3 (L) | 8.34 | 7.45 | 8.69 | 6.50 | 8.24 | 4.45 | 7.44 | 6.31 | 8.72 | 8.46 | 8.67 | 6.51 |
| T3 (R) | 8.34 | 7.9 | 8.83 | 6.38 | 8.44 | 5.42 | 7.94 | 7.23 | 8.91 | 8.62 | 8.36 | 5.40 |
| T8 (L) | 9.85 | 6.63 | 9.92 | 4.91 | 9.35 | 3.63 | 8.75 | 5.50 | 10.25 | 5.31 | 9.92 | 4.11 |
| T8 (R) | 9.89 | 6.43 | 10.08 | 5.32 | 9.18 | 4.23 | 8.98 | 5.28 | 10.89 | 5.05 | 9.52 | 5.20 |
| Femoral N (L) | 17.70 | 6.93 | 19.21 | 8.03 | 16.42 | 4.37 | 15.23 | 4.78 | 18.30 | 6.01 | 17.61 | 6.03 |
| Femoral N (R) | 17.51 | 6.50 | 19.14 | 7.52 | 16.21 | 6.71 | 15.61 | 4.51 | 18.92 | 6.66 | 18.03 | 5.49 |
| Tibial N (L) | 19.05 | 6.38 | 21.03 | 6.26 | 17.55 | 8.13 | 17.25 | 6.43 | 20.05 | 7.01 | 19.38 | 6.51 |
| Tibial N (R) | 18.85 | 7.34 | 20.67 | 7.41 | 17.95 | 7.61 | 16.84 | 6.23 | 19.58 | 6.72 | 18.75 | 7.22 |
| Common peroneal N (L) | 19.93 | 5.90 | 21.95 | 7.21 | 18.43 | 5.98 | 18.13 | 6.65 | 21.03 | 5.96 | 20.33 | 4.95 |
| Common peroneal N (R) | 20.05 | 6.65 | 22.32 | 6.91 | 19.02 | 6.21 | 18.02 | 6.89 | 20.83 | 4.68 | 20.62 | 5.37 |

**Supplementary Table S4. The average (basic value) of SSEPs latency and amplitude of 6 animals before SCI**

“C1-C6”is the animal number; “(L)” = “Left limb”, “(R)” = “Right limb”; "L(ms)"=SSEPs latency, "A(μv)"= SSEPs amplitude


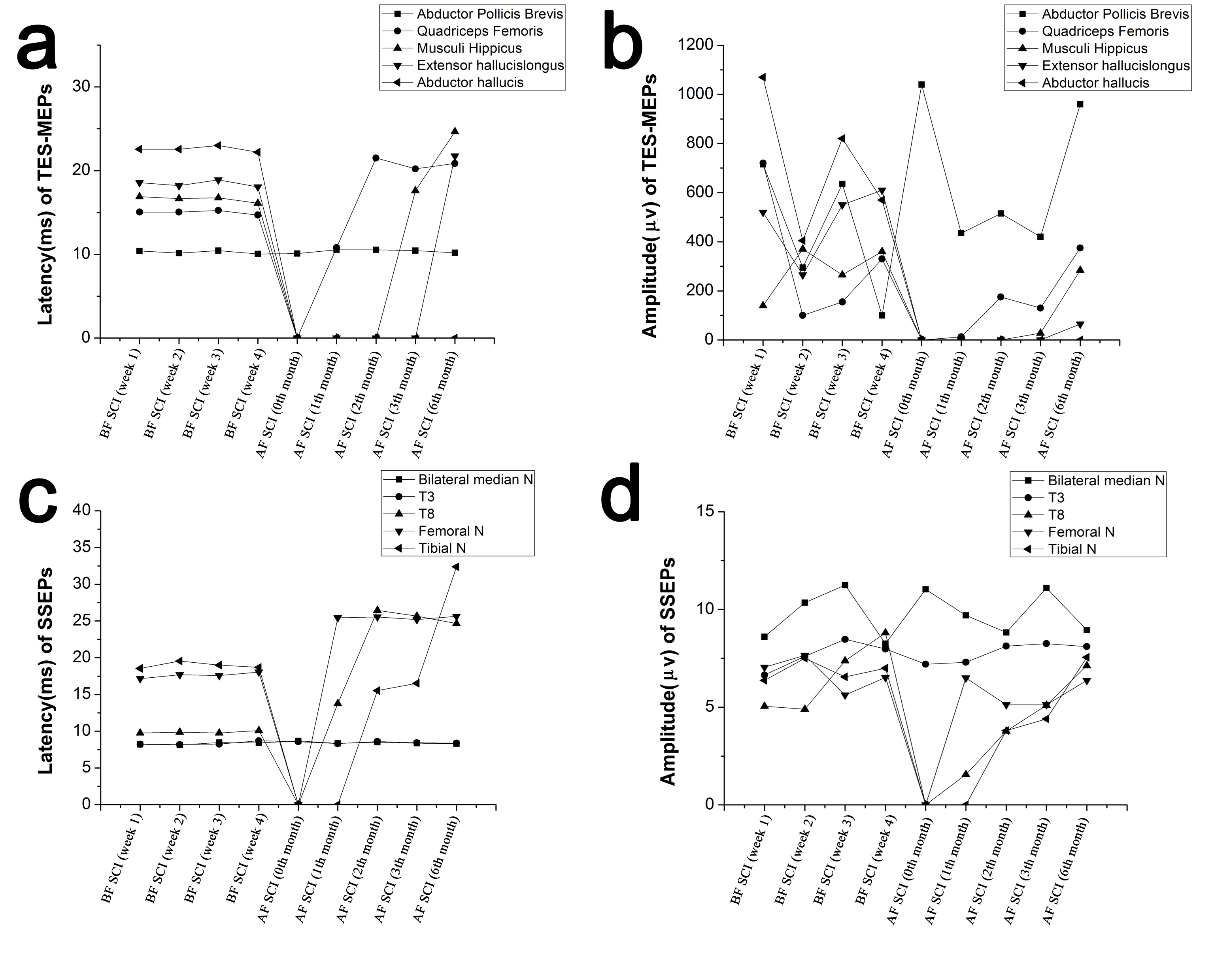


**Supplementary Figure S5. The changing process of latency and amplitude of TES-MEPs and SSEPs of NO. C1**

**
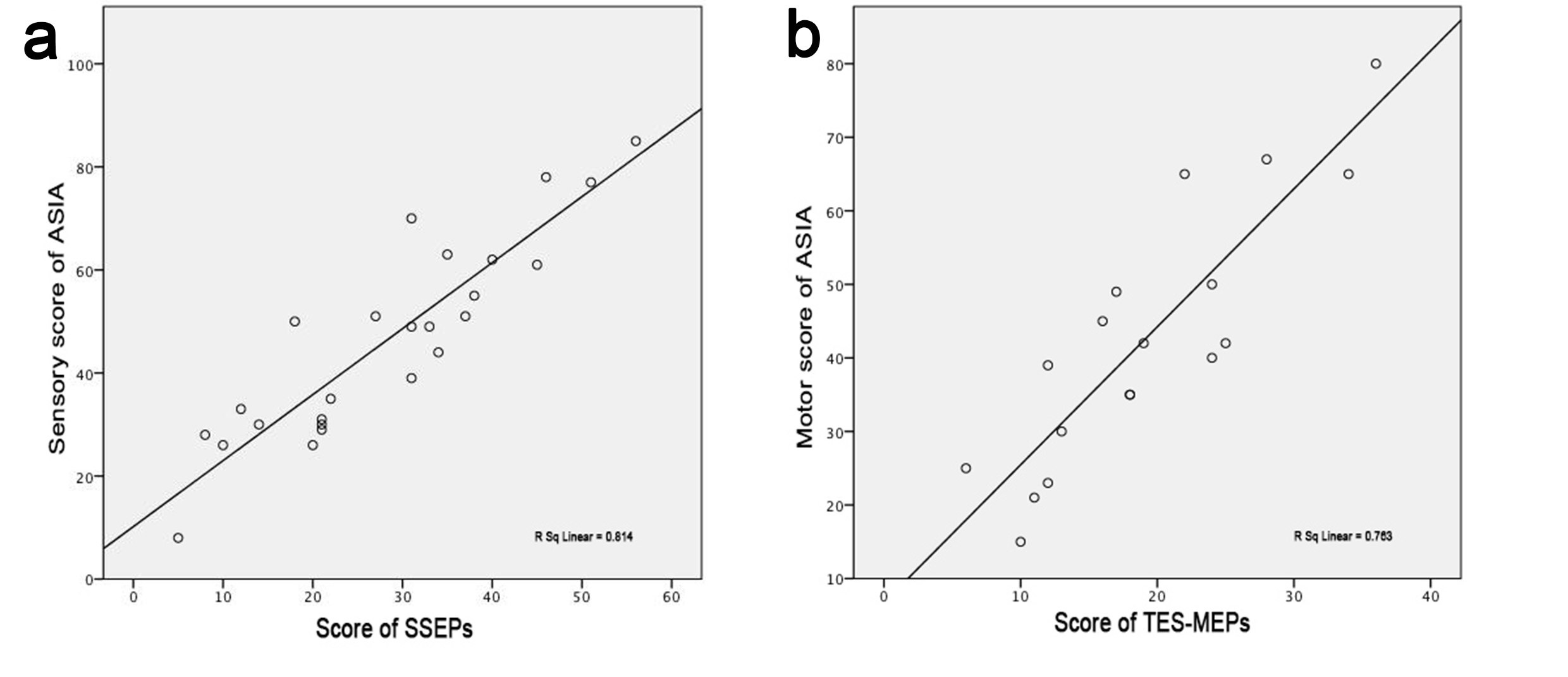
**

**Supplementary Figure S6. The correlation between EP and ASIA score in SCI patients**
